# Occurrence of HSV-1-induced pneumonitis in patients under standard immunosuppressive therapy for rheumatic, vasculitic, and connective tissue disease

**DOI:** 10.1186/1471-2466-9-22

**Published:** 2009-05-18

**Authors:** Matthias N Witt, Gerald S Braun, Stephan Ihrler, Holger Schmid

**Affiliations:** 1 Department of Rheumatology, Medical Policlinic, University of Munich, Munich, Germany; 2 Department of Nephrology and Clinical Immunology, RWTH University, Aachen, Germany; 3 Department of Pathology, University of Munich, Munich, Germany; 4 Department of Nephrology, Medical Policlinic, University of Munich, Munich, Germany

## Abstract

**Background:**

Herpes simplex virus type-1 (HSV-1) has been described to cause respiratory tract infections in critically ill patients or in individuals that are immunocompromised. It is a continuing matter of debate under which circumstances HSV-1 is a relevant pathogen for pneumonitis. While its role during critical illness has been investigated by prospective interventional studies, comparatively little systematic data is available on the role of HSV-1 for pneumonitis in outpatients with autoimmune disease under a maintenance regimen of immunosuppression.

**Methods:**

We retrospectively reviewed the charts of ~1400 patients with rheumatoid arthritis, vasculitis, and systemic lupus erythematosus (SLE) that were followed at the outpatient clinic of a German University hospital during the years 2000–2007. Episodes of admission to a ward resulting in the diagnosis of pneumonia/pneumonitis were identified, and the type of pneumonia and clinical features retrospectively studied.

**Results:**

63 patients with rheumatoid arthritis, vasculitis, or SLE were admitted to a ward and diagnosed to have pneumonia/pneumonitis. Using bronchoscopy a total of 6 cases of pulmonary infection associated with HSV-1 in the lower respiratory tract were identified. Among those, 2 cases suggested a causative role of HSV-1 as the sole agent causing pneumonitis that proved clinically responsive to antiviral treatment. In the remaining 4 cases HSV-1 appeared as a bystander of bacterial infection. Maintenance therapy with leflunomide, which inhibits HSV-1 assembly *in vitro*, was associated with a milder course of pneumonitis in one patient. Detection of HSV-1 was associated with stronger immunosuppressive regimens and vasculitic disease.

**Conclusion:**

The present study analyzed the frequency and hallmarks of cases of HSV-1 associated pneumonitis that occurred in a comparatively large cohort of patients with rheumatologic autoimmune diseases. In an area of controversy, this study provides further evidence that HSV-1 causes isolated pneumonitis in the immunocompromised. The study may provide an estimate on the frequency of relevant HSV-1 infection and bacterial agents in outpatients with autoimmune disease.

## Background

After reactivation from latency in facial nerve ganglia, herpes simplex virus type-1 (HSV-1) may cause tracheobronchitis or pneumonitis featuring life-threatening respiratory-failure with bilateral changes on thoracic imaging [1-4]. HSV-1 pneumonitis is comparatively well investigated in critically ill patients under mechanical ventilation, where ~90% show an oropharyngeal reactivation of the virus within the first 10 days [5]. In a number of retro- and prospective studies in this setting presence of cytology-proven HSV-1 pneumonitis was shown to have a poorer outcome, but antiviral treatment was not beneficial [6-9]. Immunosuppressed patients with malignancies, organ transplants, and cytotoxic treatments for various reasons are the other large, though extremely heterogeneous, group of patients that have been described to develop HSV-1 pneumonitis. Data on the disease in these subjects is more scarce and mainly consists of anecdotal accounts [10-16]. Only one retrospective study of bronchoalveolar lavage (BAL) microbiology/virology results from renal transplant patients is available and, very recently, a retrospective study on HSV-1 pneumonitis in patients with solid tumors was published representing the first systematic approaches in the field [17,18]. In view of lack of data, it was the goal of the present study to investigate the potential significance of HSV-1 pneumonitis in patients under a maintenance regimen of ambulatory immunosuppression. We therefore retrospectively analyzed admissions for pneumonia/pneumonitis in a comparatively large cohort of outpatients with rheumatoid, vasculitic and connective tissue autoimmune disease over a period of 8 years.

## Methods

This retrospective study was approved by the local Ethics Committee. The local electronic database containing all patient diagnoses and discharge summaries of our rheumatologic outpatient clinic and the affiliated hospitals of the Department of internal medicine were searched for a combination of the terms of the rheumatologic/vasculitic/connective tissue disease and pneumonia or pneumonitis. Each episode of a positive hit was reviewed. Severity of current immunosuppressive treatment was scored 0–4 as follows: 0 = no immunosuppressive medication; 1 = low-dose: (prednisone ≤ 10 mg/d) or (azulfidine/chloroquine); 2 = intermediate: (prednisone 20–40 mg/d) or (prednisone 10 mg/d+methotrexate) or (prednisone 10 mg/d+leflunomide) or (cyclosporine) or (prednisone+mycophenolate mofetil) or (prednisone 20 mg/d+azathioprine); 3 = strong: (prednisone 40 mg/d+methotrexate) or (prednisone 20 mg/d+methotrexate+anakinra) or (prednisone 20 mg/d+methotrexate+leflunomide) or (prednisone 20 mg/d+etanercept) or (prednisone 40 mg/d+azathioprine) or (lower dose prednisone+azathioprine+leukopenia); 4 = maximal: (prednisone 60 mg/d+cyclophosphamide) or (prednisone 100 mg/d).

At the time of in-hospital treatment the patients were diagnosed and treated by current standards of clinical practice and were not part of a prospective clinical trial regarding the treatment of pneumonia or pneumonitis.

Fiberoptic bronchoscopy (FOB) was performed when deemed appropriate. Bronchoalveolar lavage (BAL) was performed with each bronchoscopic examination and material was subsequently centrifuged, fixated in 4% paraformaldehyde and stained using hematin-eosin. Standard microscopic examination was performed. BAL fluids were also subjected to viral polymerase chain reaction (PCR) when deemed appropriate that always consisted of testing for HSV-1 and CMV (cytomegaly virus). In selected positive cases immunohistochemistry of BAL for HSV-1 was performed using a rabbit polyclonal antibody against HSV-1 (DAKO Laboratories, Denmark). Bronchoscopic biopsies were performed when deemed appropriate and processed using standard protocols. Microbiologic and serologic diagnostics were performed using standard protocols.

Statistical analysis was performed using Prism 4 software (Graph Pad, La Jolla, CA).

## Results

As it is unclear to which extent an outpatient regimen of current standard immunosuppression for autoimmune disease may confer a risk for HSV-1 pneumonitis, we retrospectively reviewed the charts of ~1400 patients with rheumatoid arthritis (~1000), ANCA-associated vasculitis (~80), and systemic lupus erythematosus (SLE) (~320) that were followed during the years 2000–2007 in the nephrology and rheumatology outpatient clinic at the Medical Policlinic, LMU University of Munich. During the study period 766 ward-admissions of patients with rheumatologic/vasculitic/connective tissue autoimmune disease occurred. Among those there were 63 (8%) episodes of admission from 63 outpatients of our clinic presenting with respiratory deterioration that were ultimately diagnosed to be due to pneumonia or pneumonitis (Table 1). As a rough estimate one can infer an incidence of community-acquired respiratory infection or pneumonitis requiring hospitalization in outpatients with autoimmune disease of [63 patients * 100/(1400 patients *8 years)], i.e., ~0.5% per year.

**Table 1:** Classification and clinical course of the study population, i.e., 63 patients with rheumatic/vasculitic or connective tissue disease admitted to hospital and diagnosed to have pneumonia/pneumonitis

	**Total admissions to hospital**	**Admissions for PNA (% of admissions)**	**FOB with BAL in PNA (% of PNA)**	**BAL-PCR for HSV-1 and CMV (% of FOB)**	**Positive HSV-1 BAL-PCR (% of PNA)**	**Requirement of intubation in PNA (% of PNA)**	**Mean and (median) age of patients with PNA in ys**	Mean immuno-suppression severity score of patients with PNA*	**Lethal outcome in PNA **
**Wegener's Granulomatosis**	74	13 (18%)	4	4	2 (cases 3, 5)	2	66 (68)	3.3	2
**Rheumatoid arthritis**	515	34 (7%)	12	6	2 (cases 1,4)	5	71 (74)	1.5	3
									
**Polyangiits**	22	4 (18%)	3	1	1 (case 2)	0	63 (64)	3.5	0
**Systemic lupus erythematosus (SLE)**	155	12 (8%)	4	3	1 (case 6)	1	53 (58)	1.6	1
									
**total**	**766**	**63 (8%)**	**23 (37%)**	**14 (61%)**	**6 (10%)**	**8 (12%)**	**66 (66)**	**2.0**	**6 (10%)**

Absolute numbers or percentages are given, (*) based on 56 of the 63 cases where information on immunosuppressive regimens was reliably available. In order to estimate the degree of immunosuppressive strength in the patient population, an arbitrary score ranging form 0–4 was applied (see methods). Abbreviations: BAL = bronchoalveolar lavage, FOB = fiberoptic bronchoscopy, PNA = pneumonia/pneumonitis, ys = years.

In 23 of these 63 patients bronchoscopy with lavage was performed for diagnostic purposes within the first four days of admission, which in 14 (61%) also comprised PCR analysis for HSV-1. Thereby a total of six patients (10% of admissions for pneumonia) with positive HSV-1 DNA detection in alveolar fluids was identified. In order to estimate the degree of immunosuppressive strength in the patient population, an arbitrary score ranging form 0–4 was applied (see methods).

Relevant clinical, imaging and laboratory data on the six patients with a positive HSV-1 DNA detection on BAL are summarized in Table 2.

**Table 2:** Synopsis of patient data on all cases with a positive HSV-1 result on PCR of bronchoalveolar lavage

**Clinical data**	**Case 1**	**Case 2**	**Case 3**	**Case 4**	**Case 5**	**Case 6**
Age at presentation (yrs), gender	74, f	74, m	60, m	67, m	72, m	65, f
Rheumatological diagnosis (duration in years)	Rheumatoid arthritis, seronegative 8	pulmonary microscopic Polyangiitis 3	Wegener's Granulomatosis 2	Rheumatoid arthritis, seropositive 10	Wegener's Granulomatosis 8	Systemic lupus erythematosus 4
Co-Morbidities	allergic asthma		renal insufficiency	diabetes, septic arthritis	HHT, renal insufficiency	
Immunosuppression (severity score)	Pred 20 mg/d + MTX 15 mg/w + Lefl 20 mg/d (3)	Pred 50 mg/d + Cyclo 150 mg/d p.o. (4)	Pred 20 mg/d +Cyclo 150 mg/d (4)	Pred 20 mg/d + MTX 7.5 mg/w + Anakinra 100 mg/d (3)	Pred 40 mg/d + Cyclo 500 mg/m i.v. (4)	Pred 20 mg/d + Aza 150 mg/d, pancytopenia (3)
clinical presentation in the outpatient setting prior to admission	21 days of cough, fever	3 days of bloody cough, dysphagia	3 days of dyspnea, syncope	7 days of dyspnea, cough	5 days of dyspnea, cough, weakness	1 day of dyspnea, non-productive cough
Notable findings	HSV-1 positive oral lesion	none	HSV-1 positive oral lesion	none	HSV-1 positive nasal lesion	none
Ventilatory support required	CPAP	no	intubation	intubation	no	no
**Radiological findings**						
Chest-XR	diffuse bilateral ground-glass opacities	interstitial pattern	interstitial pattern	bilateral bronchopneumonic infiltrates with pleural effusions	bilateral bronchopneumonic infiltrates	unilateral infiltrate
High-resolution CT-scan	diffuse bilateral ground-glass opacities	right-sided diffuse pleural effusions	diffuse bilateral ground-glass opacities (no granulomas)	extensive bilateral bronchopneumonic infiltrates with pleural effusions, atelectasis, hilar lymphadenopathy	diffuse bilateral granulomas	unilateral bronchopneumonic infiltrate
**Bronchoscopy/pathology**						
performed on hospital day	2	2	2	2	2	4
Macroscopic mucosal aspect	Vulnerable, inflammamation	normal	inflammation	acute bronchitis	vulnerable, acute bronchitis	vulnerable
BAL cytology: inclusion bodies	Positive	positive	positive	n.a.	n.a.	n.a.
Lung biopsy	Non-specific (chronic) bronchitis	n.p.	non-specific fibroelastosis	n.p.	n.p.	n.p.
HSV-1 immunohistology of BAL cytology or of lung biopsy	n.p.	positive	n.p.	n.p.	n.p.	n.p.
**Relevant identified infectious agents of pneumonia/pneumonitis (from BAL)**	**HSV-1 as sole agent**	**HSV-1 as sole agent**	Aspergillus, K. pneumoniae, HSV-1	MRSA, P. aeruginosa, K. pneumoniae, HSV-1	S. aureus, P. aeruginosa, Influenza A, HSV-1	S. aureus (coag neg), P. aeruginosa, Enterococci, HSV-1, M. kansasii
**Treatment**	- day 1: ceftriax+ery (for 7 days; overlap with acyclo 5 days) - day 2: acyclo	- day 1: moxi (for 2 days) - day 2: swap to acyclo monotherapy	- day 1: mero + ery + fluc - day 2: add acyclo + amphoB for fluc - later: add cipro+ tobra + vanco for mero	sequential: - ceftr+moxi - genta+tazo+fluc - mero - vanc+linez from day 2: acyclo	sequential: - metro+clari +ceftriax - day 3 ceftaz for ceftriax	sequential: - clari+cipro+ceftaz - fluc (for 14 days) from day 14: INH+ rifampicin+ethambutol
**Outcome**	recovery after 7 days	recovery after 11 days	lethal after 28 days (ARDS)	lethal after 33 days (ARDS)	Initial recovery after 28 days, but lethal after 110 days (ARDS) with persistant high HSV-1 viral load on BAL	recovery after 38 days
**Our Diagnosis/comment**	most likely isolated HSV-1 pneumonitis	most likely isolated HSV-1 pneumonitis	Bacterial, fungal and HSV-1 pneumonia/pneumonits	Bacterial bronchopneumonia with HSV-1 reactivation	Bacterial pneumonia with untreated HSV-1 reactivation. A second BAL PCR showed an increasing viral load	Mycobacteriosis due to M. kansasii (responsive to treatment). HSV-1 reactivation without radiographic signs of pneumonitis that was not treated antivirally.
**Complete microbiological work-up**						
Blood cultures	negative	negative	negative	negative	negative	negative
Mycobacteria culture/PCR BAL	negative	negative	negative	negative	negative	**positive **(M. kansasii)
Aspergillus	negative	negative	**positive **(serum Ag & culture)	negative	n.p.	negative (serum AG)
P. jirovecii BAL	negative	negative	negative	negative	negative	negative
MRSA culture Sputum	negative	negative	negative	**positive**	negative	negative
P. aeruginosa culture BAL	negative	negative	negative	**positive**	**positive**	**positive**
K. pneumoniae culture BAL	negative	negative	**positive**	**positive**	negative	negative
M. pneumoniae	negative (PCR BAL)	negative (serology)	negative (serology)	negative (PCR BAL)	negative (PCR BAL)	n.p.
C. trachomatis/pneumoniae	negative (serology)	n.p.	negative (PCR BAL)	negative (PCR BAL)	negative (PCR BAL)	negative (PCR BAL)
Legionella-Ag (Urine)	negative	negative	negative	negative	negative	n.p.
Complete virological work-up						
HSV-1 PCR BAL, Geq/ml	9.750.000	284.000	700.000	850.000	10.250.000	310.000
CMV-PCR BAL	negative	Negative	negative	negative	negative	negative
Influenza A PCR BAL* (season of presentation)	n.p. (May)	n.p. (April)	negative (January)	n.p. (September)	**positive **(February)	n.p. (June)
Adenovirus PCR BAL**	Negative	negative	negative	n.p.	n.p.	n.p.
HIV-1/2-Ag ELISA	n.p.	n.p.	negative	n.p.	Negative	n.p.

Abbreviations used are: acyclo = acyclovir, Ag = antigen, amphoB = amphotericin B, ARDS = adult respiratory distress syndrome, Aza = azathioprine, BAL = bronchoalveolar lavage, ceftaz = ceftazidime, ceftriax = ceftriaxone, cipro = ciprofloxacin, clari = clarithromycin, CPAP = continuous positive airway pressure, CT = computed tomography, coag neg = coagulase negative, cyclo = cyclophosphamide, d = day, ery = erythromycin, fluc = fluconazole, genta = gentamycin, Geq/ml = genome equivalents/ml; HHT = hereditary hemorrhagic telangiectasia (Osler-Weber-Rendu syndrome), INH = isoniazid, Lefl = leflunomide, linez = linezolid, mero = meropenem, metro = metronidazole, moxi = moxifloxacin, MTX = methotrexate, Pred = prednisone, tazo = piperacillin/tazobactam, tobra = tobramycin, vanco = vancomycin, w = week. *testing was seasonal during winter only. **testing was at the discretion of the physician performing bronchoscopy.

**Figure 1:**
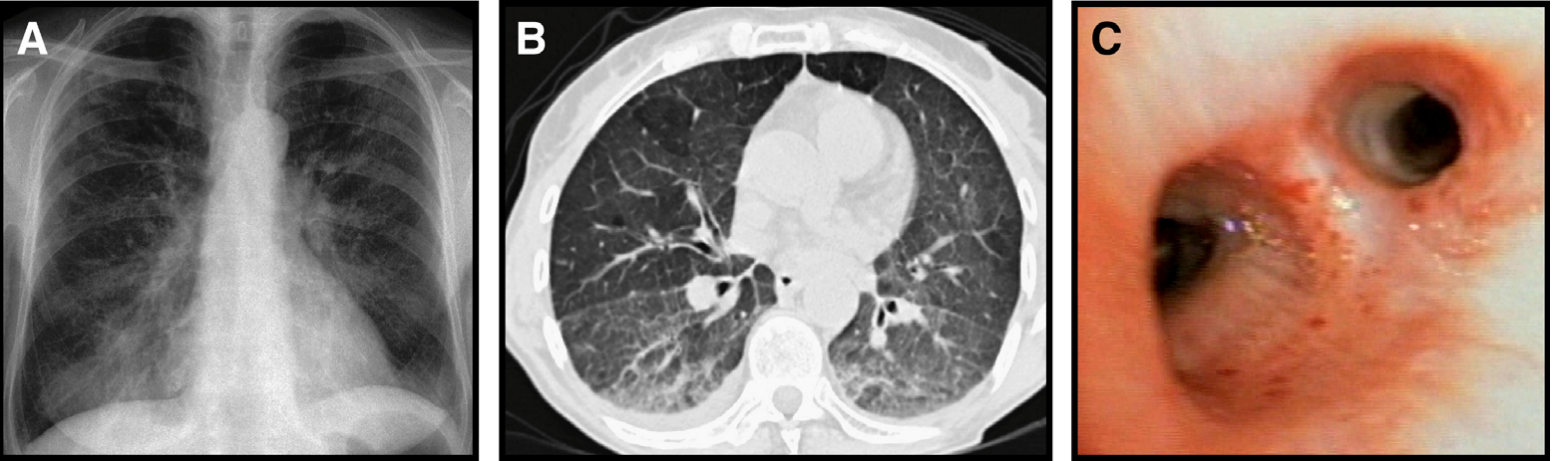
**Imaging study from Case 1**. A: Conventional chest radiograph with diffuse interstital pattern. B: High-resolution-CT (HRCT) featuring small pleural effusions and partial atelectasis of the right lower lobe in addition to distinctive ground-glass opacities. C: Fiberoptic bronchoscopy image showing a vulnerable tracheobronchial mucosa with multiple spontaneous bleeding stigmata, consistent with generalized tracheobronchial inflammation.

**Figure 2:**
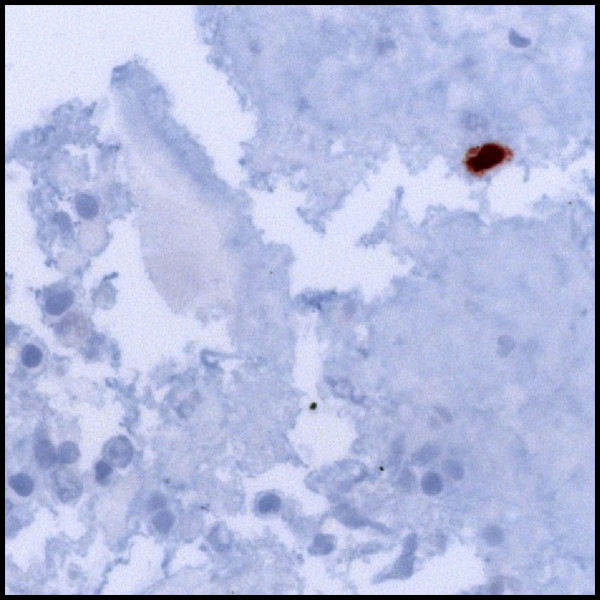
**Immunohistochemical staining for HSV-1 in a fixated cyotspun obtained from BAL from Case 2 identifying a positive cell (dark brown colour)**. Bottom and left: alveolar macrophages and lymphocytes with degenerative changes.

Two cases (Case 1 and 2) in which no other viral, bacterial or fungal agent than HSV-1 on BAL PCR was found and in which imaging studies were suggestive for HSV-1 pneumonitis were successfully treated with acyclovir leading to rapid clinical improvement. In both cases, the initial chest radiograph and thoracic CT showed diffuse infiltrations or ground glass opacities. Representative images are shown in Figure 1A, B. HSV-1 infection may feature a macroscopically vulnerable aspect of the bronchial mucosa as a non-specific sign (Figure 1C). In order to increase diagnostic certainty in Case 2, immunohistochemical staining of bronchioalveolar cells for HSV-1 was performed, which proved positive (Figure 2). In both cases inclusion bodies of epithelial cells from BAL were present. Our diagnosis in Case 1 and 2 was isolated HSV-1 pneumonitis, which was supported by the response to acyclovir in the absence of prolonged antibacterial treatment.

Case 3 and 4 displayed an initial co-infection with fungal or bacterial agents and HSV-1 and required prolonged mechanical ventilation. Imaging studies were compatible with viral pneumonitis, but in Case 4 infiltrates suggesting bronchopneumonia were also clearly present. The mucosa was macroscopically inflamed in both cases. While Case 3 had inclusion bodies on cytology, such studies were not pursued in Case 4 due to obvious bronchopneumonia. Both patients had a fatal outcome after a prolonged in-hospital stay despite adequate antiinfective treatment that included timely administration of acyclovir.

Cases 5 and 6 are different from the former four cases therein that no bilateral groundglass opacities were present on thoracic CT and no antiviral treatment was initiated. In Case 5, a high HSV-1 viral load from BAL fluids was ignored given the detection of Influenza A virus with S. aureus and Ps. aeruginosa. The patient improved on antibacterial treatment alone and was discharged. He was readmitted one month later with *de-novo *respiratory deterioration that resulted in the need for intubation after the administration of a series of antimicrobial courses that always excluded acyclovir. CT and a second BAL now showed ground glass opacities/ARDS and an even higher HSV-1 viral load. The patient died from ARDS before antiviral treatment could be initiated. Case 6 featured an unilateral bronchopneumonic infiltrate and was treated with standard antibacterial regimens until *Mycobacterium kansasii *grew from the initial BAL. Treatment was changed accordingly and the patient recovered without the administration of antivirals.

In an area of controversy, the first two cases provide further evidence that in selected patients, if ascertained to be the sole agent, HSV-1 is a relevant pathogen of pneumonitis that is amenable to treatment. Cases 3–6 reflect the great difficulty of dissecting the role of HSV-1 in infections with multiple pathogens [19] and illustrate that HSV-1 pneumonitis is a diagnosis of exclusion requiring a complete microbiological workup. It remains speculative if timely suppression of HSV-1 viral loads by antiviral treatment in Case 5 would have altered the patient's course.

**Figure 3:**
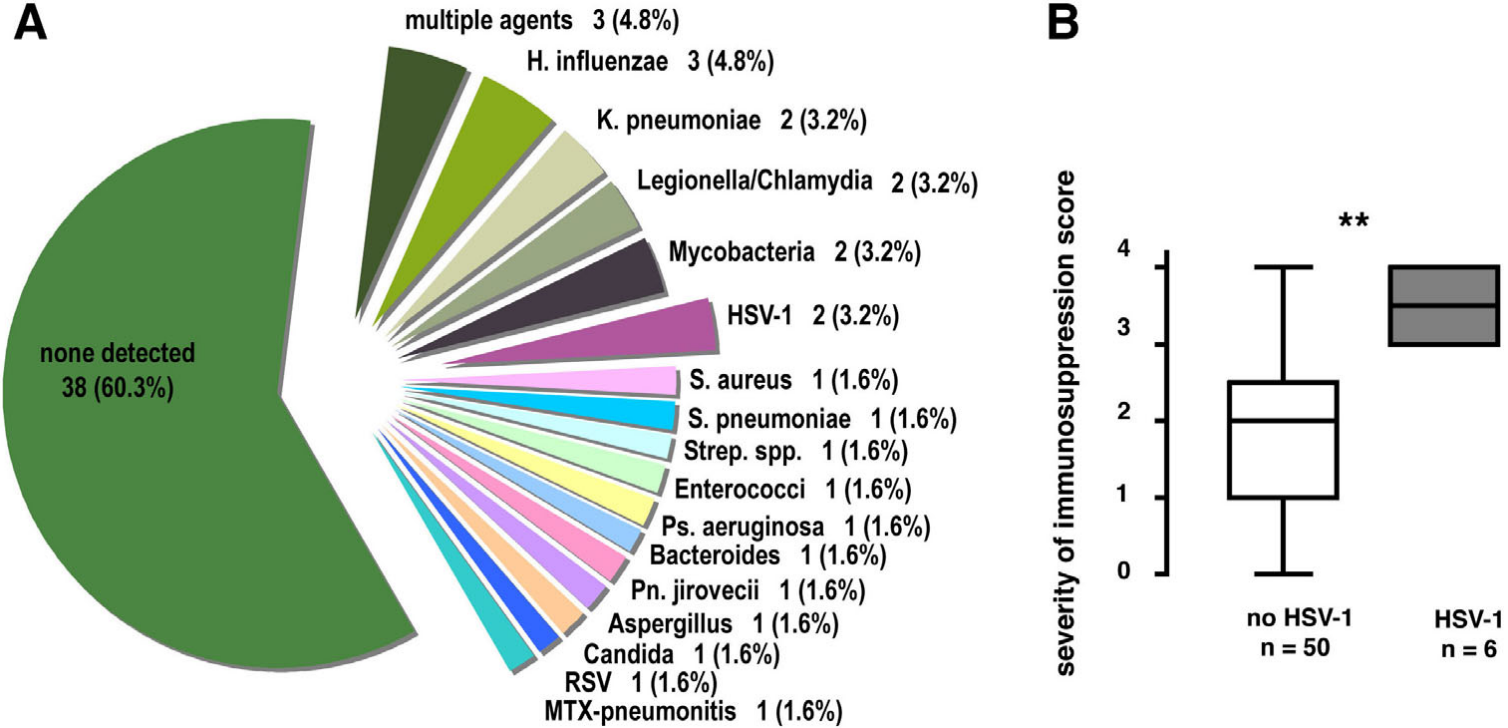
**A: Detected primary responsible (leading) infectious agents in 63 patients with ambulatory-acquired pneumonia/pneumonitis and autoimmune disease**. RSV = respiratoy syncytial virus. B: Immunosuppression scores were significantly more severe in the 6 patients with HSV-1 detection in BAL than in those subjects without clinical or laboratory evidence for HSV-1 (as assessed for 56/63 patients with reliable information on immunosuppressive regimens available; ** p < 0.01, Mann-Whitney two-sided test).

In the present study of 63 patients with autoimmune disease and pneumonia/pneumontis, no offending agent could be found in 60% of cases. In the remaining 40% a broad spectrum of bacteria, fungi and viruses was identified (Figure 3A). Of note, one case of pneumonitis was due to methotrexate. Two cases (3.2%) were most likely due to HSV-1, while HSV-1 was a bystander of other agents in the remaining four cases.

When comparing the six HSV-1-positive cases to those where HSV-1 was neither detected nor suspected with respect to the severity of immunosuppression, a significantly higher degree of immunosuppression in the HSV-1-positive group was noted (p < 0.01, two-sided Mann-Whitney analysis) (Figure 3B). This analysis was performed including 56 of the 63 patients where reliable information on maintenance immunosuppression was available. In order to exclude selection bias, the comparison was also performed using the subgroup of 8 patients that were proven to be HSV-1 negative by BAL. Albeit not reaching the same level of significance, a similar trend was observed with a similar median immunosuppression severity score of 2.0 in the HSV-1 negative group (data not shown). Though great caution must be applied in interpreting small numbers, it is intriguing that patients with Wegener's Granulomatosis and vasculitis, who had the strongest immunosuppression (score 3.3–3.5) also had a HSV-1 positive BAL more often (15–25% of pneumonia/pneumonitis) than Rheumatoid arthritis and SLE patients (6–8% of pneumonia/pneumonitis) who were also on a milder immunosuppressive regimen (score 1.5–1.6) (Table 1).

## Discussion

Patients with systemic autoimmune disease have an alteration of immune responses that is due to both the action of immunosuppressive treatment and to the underlying condition itself [20,21]. They are at increased risk for developing opportunistic infections among which respiratory tract involvement is associated with potentially adverse outcomes. Clinical workup of respiratory deterioration in this setting is complicated by the fact that not only bacterial, fungal or viral infectious agents but also a pulmonary flare of the systemic disease or drug-related pulmonary toxicity must be taken into the differential diagnosis. Indicators that raise suspicion for HSV-1 pneumonitis in immunocompromised patients are severe respiratory insufficiency combined with pathologic findings on imaging studies. Thoracic radiographs show segmental bilateral opacities (~95%) and pleural effusions (~50%) while CT may show ground-glass opacities (~100%), focal consolidations (~75%), and pleural effusions (~88%) [22,23]. Especially ground-glass opacities are a useful diagnostic hint in the immunocompromised, suggesting either an opportunistic infection due to Pneumocystis jirovecii, CMV, HSV-1, or other viruses [24]. Other non-infectious differential diagnoses of ground glass opacities have been reviewed by [24].

HSV-1 pneumonitis is a diagnosis of exclusion relying on clinical plausibility, positive viral testing, and imaging studies. The definition of clinically relevant positive viral testing is a matter of controversy. A recent study in patients under prolonged mechanical ventilation by Luyt *et al*. required the combination of (i) clinical deterioration, (ii) HSV-1 detection in the lower respiratory tract by either PCR or culture, and (iii) cytological or histological evidence of inclusion bodies from either BAL fluids or biopsies to specifically define HSV-1 pneumonitis [9]. While viral cultures are difficult to handle, PCR of BAL fluids has become the most popular diagnostic tool since it is easily performed and sports a high sensitivity. Specificity however, may be lacking due to potential contamination with fluids from oropharyngeal reactivation, which may even occur in plain bacterial pneumonia [5,9,19,25]. Indeed, in Luyt's study, only 43% of those with a positive PCR result also had cytological/histological evidence of infection. Conversely, these cytological/histological criteria lack sensitivity as illustrated by the fact that even open lung biopsies may be negative in the case of autopsy-proven HSV-1 pneumonitis [4]. From a theoretical point of view, HSV-1 viraemia could be an additional specific diagnostic tool, which is, again, associated with a marked reduction in sensitivity [9].

A careful microbiological and remaining virological workup is necessary to define by exclusion isolated HSV-1 pneumonitis. Unfortunately, results of bacterial sampling are often falsely negative, be it due to inadequately low amounts material, assay insensitivity or prior antibiotic use, to name just a few. This is also illustrated by the 60% of cases of pneumonia in our study in which no offending agent was detected despite clinical evidence of infection. Hence, the possibility of falsely negative bacterial testing also applies as a *caveat *to our Cases 1 and 2. Our centre performed viral testing of BAL fluids for influenza routinely during winter. Concerning other viruses such as adenovirus and coronavirus each centre should define a standard in order to avoid omission or over-testing.

Viral load of BAL fluids detected by PCR correlates positively with the presence of cytology/histology-proven HSV-1 pneumonitis and negatively with outcome [6,9,16], but is not necessarily helpful in establishing the diagnosis due to potentially great variations in sampling conditions. However, it is intriguing that Case 1 in our series who was on treatment with leflunomide showed a viral load (9.750.000 Geq/ml) that was 3 and 13-fold greater than the detected average (3.700.000) and median (775.000) of all cases, respectively, while the clinical course was prolonged (21 days prior to admission to hospital) and comparatively mild. Leflunomide exhibits antiviral effects against HSV-1 by inhibiting the assembly of viral capsids but not DNA-replication at dosages used in rheumatic patients [26]. It is tempting to speculate, that leflunomide might have led to the shedding of ill-assembled virions in Case 1.

As outlined in the introduction, studies on HSV-1 pneumonitis in patients with various immunosuppressive conditions are just beginning to emerge [27]. The present study retrospectively analyzed admissions for pneumonia/pneumonitis in a comparatively large cohort of outpatients with autoimmune disease under a maintenance regimen of immunosuppression. Although the considerable limitations of a retrospective design must be borne in mind, the data provide some perspective on the frequency of HSV-1 and other bacterial, fungal and viral agents leading to pulmonary infection in this patient group.

## Conclusion

Respiratory deterioration and pneumonia leading to hospital admission was a common event in outpatients with autoimmune diseases treated with current standard immunosuppressive regimens. Acute life-threatening respiratory failure associated with the detection of HSV-1 in the lower respiratory tract was a rare, but significant finding. Based on our findings, diagnostic virological workup in this particular patient cohort should include HSV-1 before starting, but without delaying treatment. In an area of controversy [19,28], we provide further evidence that immunocompromised patients may develop a condition that is most accurately described as HSV-1 pneumonitis and that appears amenable to treatment.

## Competing interests

The authors have no affiliation or significant financial involvement in any organizations or entity with a direct financial interest in the subject matter or materials discussed in the manuscript. This includes employment, honoraria, consultancies, or relevant stock ownership.

## Authors' contributions

MNW carried out the retrospective chart review, analyzed the data and helped to draft the manuscript. GSB drafted the manuscript including figures and tables and participated substantially in the coordination of the study and the analysis of data. SI processed the cytological samples from BAL and evaluated the lung biopsies. HS had the idea for the study, participated in its design and coordination and helped to draft the manuscript. All authors read and approved the final manuscript. MNW and GSB contributed equally and are both considered first authors.

## Pre-publication history

The pre-publication history for this paper can be accessed here:


